# Stability of the stationary solutions of neural field equations with propagation delays

**DOI:** 10.1186/2190-8567-1-1

**Published:** 2011-05-03

**Authors:** Romain Veltz, Olivier Faugeras

**Affiliations:** 1 IMAGINE/LIGM, Université Paris Est., France; 2 NeuroMathComp team, INRIA, CNRS, ENS Paris, France

## Abstract

In this paper, we consider neural field equations with space-dependent delays. Neural fields are continuous assemblies of mesoscopic models arising when modeling macroscopic parts of the brain. They are modeled by nonlinear integro-differential equations. We rigorously prove, for the first time to our knowledge, sufficient conditions for the stability of their stationary solutions. We use two methods 1) the computation of the eigenvalues of the linear operator defined by the linearized equations and 2) the formulation of the problem as a fixed point problem. The first method involves tools of functional analysis and yields a new estimate of the semigroup of the previous linear operator using the eigenvalues of its infinitesimal generator. It yields a sufficient condition for stability which is independent of the characteristics of the delays. The second method allows us to find new sufficient conditions for the stability of stationary solutions which depend upon the values of the delays. These conditions are very easy to evaluate numerically. We illustrate the conservativeness of the bounds with a comparison with numerical simulation.

## 1 Introduction

Neural fields equations first appeared as a spatial-continuous extension of Hopfield networks with the seminal works of Wilson and Cowan, Amari [1,2]. These networks describe the mean activity of neural populations by nonlinear integral equations and play an important role in the modeling of various cortical areas including the visual cortex. They have been modified to take into account several relevant biological mechanisms like spike-frequency adaptation [3,4], the tuning properties of some populations [5] or the spatial organization of the populations of neurons [6]. In this work we focus on the role of the delays coming from the finite-velocity of signals in axons, dendrites or the time of synaptic transmission [7,8]. It turns out that delayed neural fields equations feature some interesting mathematical difficulties. The main question we address in the sequel is that of determining, once the stationary states of a non-delayed neural field equation are well-understood, what changes, if any, are caused by the introduction of propagation delays? We think this question is important since non-delayed neural field equations are pretty well understood by now, at least in terms of their stationary solutions, but the same is not true for their delayed versions which in many cases are better models closer to experimental findings. A lot of work has been done concerning the role of delays in waves propagation or in the linear stability of stationary states but except in [9] the method used reduces to the computation of the eigenvalues (which we call *characteristic values*) of the linearized equation in some analytically convenient cases (see [10]). Some results are known in the case of a finite number of neurons [11,12] and in the case of a few number of distinct delays [13,14]: the dynamical portrait is highly intricated even in the case of two neurons with delayed connections.

The purpose of this article is to propose a solid mathematical framework to characterize the dynamical properties of neural field systems with propagation delays and to show that it allows us to find sufficient delay-dependent bounds for the linear stability of the stationary states. This is a step in the direction of answering the question of how much delays can be introduced in a neural field model without destabilization. As a consequence one can infer in some cases without much extra work, from the analysis of a neural field model without propagation delays, the changes caused by the finite propagation times of signals. This framework also allows us to prove a linear stability principle to study the bifurcations of the solutions when varying the nonlinear gain and the propagation times.

The paper is organized as follows: in section 2 we describe our model of delayed neural field, state our assumptions and prove that the resulting equations are well-posed and enjoy a unique bounded solution for all times. In section 3 we give two different methods for expressing the linear stability of stationary cortical states, i.e. of the time independent solutions of these equations. The first one, section 3.1, is computationally intensive but accurate. The second one, section 3.2, is much lighter in terms of computation but unfortunately leads to somewhat coarse approximations. Readers not interested in the theoretical and analytical developments can go directly to the summary of this section. We illustrate these abstract results in section 4 by applying them to a detailed study of a simple but illuminating example.

## 2 The model

We consider the following neural field equations defined over an open *bounded *piece of cortex and/or feature space Ω ⊂ **R***^d^*. They describe the dynamics of the mean membrane potential of each of *p *neural populations.(1)

We give an interpretation of the various parameters and functions that appear in (1).

Ω is a *finite *piece of cortex and/or feature space and is represented as an open bounded set of **R***^d^*. The vectors **r **and  represent points in Ω.

The function *S *: **R **→ (0, 1) is the normalized sigmoid function:(2)

It describes the relation between the firing rate *ν_i _*of population *i *as a function of the membrane potential, e.g, *V_i _*: *ν_i _*= *S*[*σ_i _*(*V_i _- h_i_*)]. We note **V **the *p*-dimensional vector (*V*_1_, ⋯, *V_p_*).

The *p *functions *ϕ_i_*, *i *= 1, ⋯, *p *represent the initial conditions, see below. We note *ϕ *the *p*-dimensional vector (*ϕ*_1_, ⋯, *ϕ_p_*).

The *p *functions , *i *= 1>, ⋯, *p *represent external currents from other cortical areas. We note **I***^ext ^*the *p*-dimensional vector .

The *p *× *p *matrix of functions **J **= {*J*_*ij*_}_*i,j *= 1, ⋯, *p *_represents the connectivity between populations *i *and *j*, see below.

The *p *real values *h_i_*, *i *= 1, ⋯, *p*, determine the threshold of activity for each population, i.e. the value of the membrane potential corresponding to 50% of the maximal activity.

The *p *real positive values *σ_i_*, *i *= 1, ⋯, *p *determine the slopes of the sigmoids at the origin.

Finally the *p *real positive values *l_i_*, *i *= 1, ⋯, *p*, determine the speed at which each membrane potential decreases exponentially toward its rest value.

We also introduce the function

**S **: **R***^p ^*→ **R***^p^*, defined by **S**(**x**) = [*S*(*σ*_1_(*x*_1 _- *h*_1_)), ⋯, *S*(*σ_p _*(*x_p _*- *h_p_*))], and the diagonal *p *× *p *matrix **L**_0 _= diag (*l*_1_, ⋯, *l_p_*).

A difference with other studies is the intrinsic dynamics of the population given by the linear response of chemical synapses. In [9,15],  is replaced by  to use the alpha function synaptic response.

We use  for simplicity although our analysis applies to more general intrinsic dynamics, see proposition 3.10 in section 3.1.3.

For the sake of generality, the propagation delays are not assumed to be identical for all populations, hence they are described by a matrxsix  whose element  is the propagation delay between population *j *at  and population *i *at **r**. The reason for this assumption is that it is still unclear from physiology if propagation delays are independent of the populations. We assume for technical reasons that ***τ ***is continuous, i.e. . Moreover biological data indicate that ***τ ***is not a symmetric function (i.e. ), thus no assumption is made about this symmetry unless otherwise stated.

In order to compute the right-hand side of (1), we need to know the voltage **V **on some interval [-*T*, 0].

The value of *T *is obtained by considering the maximal delay:

Hence we choose *T *= *τ_m_*.

### 2.1 The propagation-delay function

What are the possible choices for the propagation-delay function ? There are few papers dealing with this subject. Our analysis is built upon [16]. The authors of this paper study, inter alia, the relationship between the path length along axons from soma to synaptic buttons versus the Euclidean distance to the soma. They observe a linear relationship with a slope close to one. If we neglect the dendritic arbor, this means that if a neuron located at **r **is connected to another neuron located at , the path length of this connection is very close to , in other words, axons are straight lines. According to this, we will choose in the following:

where *c *is the inverse of the propagation speed.

### 2.2 Mathematical framework

A convenient functional setting for the non-delayed neural field equations (see [17-19]) is to use the space  which is a Hilbert space endowed with the usual inner product:

To give a meaning to (1), we define the *history space * with , which is the Banach phase space associated with equation (3) below. Using the notation **V***_t_*(*θ *) = **V**(*t *+*θ *), *θ *∈ [-*τ_m_*, 0], we write (1) as:(3)

where

is the linear continuous operator satisfying (the notation |||·||| is defined in definition A.2 of appendix A) . Notice that most of the papers on this subject assume Ω infinite, hence requiring *τ_m _*= ∞. This raises difficult mathematical questions which we do not have to worry about, unlike [9,15,20-24].

We first recall the following proposition whose proof appears in [25]

**Proposition 2.1**. *If the following assumptions are satisfied:*

*1*. **J **∈ **L**^2^(Ω^2^,ℝ^*p*×*p*^)

*2. the external current *

*3*. ,

*Then for any *, there exists a unique solution *to (3)*.

Notice that this result gives existence on **R**_+_, finite-time explosion is impossible for this delayed differential equation. Nevertheless, a particular solution could grow indefinitely, we now prove that this cannot happen.

### 2.3 Boundedness of solutions

A valid model of neural networks should only feature bounded membrane potentials. We find a bounded attracting set in the spirit of our previous work with non-delayed neural mass equations. The proof is almost the same as in [19] but some care has to be taken because of the delays.

**Theorem 2.2**. *All the trajectories of the equation (3) are ultimately bounded by the same constant R (see the proof) if *.

*Proof*. Let us define as

We note *l *= min_*i *= 1⋯*p *_*l*_*i *_and from lemma B.2 (see appendix B.1):

Thus, if .

Let us show that the open ball of  of center 0 and radius *R*, *B_R_*, is stable under the dynamics of equation (3). We know that **V**(*t*) is defined for all *t *≥ 0s and that *f <*0 on ∂*B_R_*, the boundary of *B_R_*. We consider three cases for the initial condition **V**_0_.

If  and set . Suppose that *T *∈ **R**, then **V**(*T*) is defined and belongs to , the closure of *B_R_*, because  is closed, in effect to ∂*B_R_*. We also have  because **V**(*T*) ∈ ∂*B_R_*. Thus we deduce that for *ε *> 0 and small enough, **V**(*T *+*ε *) ∈  which contradicts the definition of *T *. Thus T ∉ **R **and  is stable.

Because *f <*0 on ∂*B_R_*, **V**(0) ∈ ∂*B_R _*implies that ∀*t >*0, **V**(*t*) ∈ *BR*.

Finally we consider the case . Suppose that ∀*t >*0, , then ∀*t >*0, , thus  is monotonically decreasing and reaches the value of *R *in finite time when **V**(*t*) reaches ∂*B_R_*. This contradicts our assumption. Thus ∃*T *> 0 |**V**(*T*) ∈ *B_R_*.   □

## 3 Stability results

When studying a dynamical system, a good starting point is to look for invariant sets. Theorem 2.2 provides such an invariant set but it is a very large one, not sufficient to convey a good understanding of the system. Other invariant sets (included in the previous one) are stationary points. Notice that delayed and non-delayed equations share exactly the same stationary solutions, also called persistent states. We can therefore make good use of the harvest of results that are available about these persistent states which we note **V***^f^*. Note that in most papers dealing with persistent states, the authors compute one of them and are satisfied with the study of the local dynamics around this particular stationary solution. Very few authors (we are aware only of [19,26]) address the problem of the computation of the whole set of persistent states. Despite these efforts they have yet been unable to get a complete grasp of the global dynamics. To summarize, in order to understand the impact of the propagation delays on the solutions of the neural field equations, it is necessary to know all their stationary solutions and the dynamics in the region where these stationary solutions lie. Unfortunately such knowledge is currently not available. Hence we must be content with studying the local dynamics around *each *persistent state (computed for example with the tools of [19]) with and without propagation delays. This is already, we think, a significant step forward toward understanding delayed neural field equations.

From now on we note **V***^f ^*a persistent state of (3) and study its stability.

We can identify at least three ways to do this:

1. to derive a Lyapunov functional,

2. to use a fixed point approach,

3. to determine the spectrum of the infinitesimal generator associated to the linearized equation.

Previous results concerning stability bounds in delayed neural mass equations are "absolute" results that do not involve the delays: they provide a sufficient condition, independent of the delays, for the stability of the fixed point (see [15,20-22]). The bound they find is similar to our second bound in proposition 3.13. They "proved" it by showing that if the condition was satisfied, the eigenvalues of the infinitesimal generator of the semi-group of the linearized equation had negative real parts. This is not sufficient because a more complete analysis of the spectrum (e.g., the essential part) is necessary as shown below in order to proof that the semi-group is exponentially bounded. In our case we prove this assertion in the case of a bounded cortex (see section3.1). To our knowledge it is still unknown whether this is true in the case of an infinite cortex.

These authors also provide a delay-dependent sufficient condition to guarantee that no oscillatory instabilities can appear, i.e., they give a condition that forbids the existence of solutions of the form *e*^*i*(**k**·**r**+*ωt*)^. However, this result does not give any information regarding stability of the stationary solution. We use the second method cited above, the fixed point method, to prove a more general result which takes into account the delay terms. We also use both the second and the third method above, the spectral method, to *prove *the delay-independent bound from [15,20-22]. We then evaluate the conservativeness of these two sufficient conditions. Note that the delay-independent bound has been correctly derived in [25] using the first method, the Lyapunov method. It might be of interest to explore its potential to derive a delay-dependent bound.

We write the linearized version of (3) as follows. We choose a persistent state **V***^f ^*and perform the change of variable **U **= **V**-**V***^f^*. The linearized equation writes(4)

where the linear operator  is given by

It is also convenient to define the following operator:

### 3.1 Principle of linear stability analysis via characteristic values

We derive the stability of the persistent state **V***^f ^*(see [19]) for the equation (1) or equivalently (3) using the spectral properties of the infinitesimal generator. We prove that if the eigenvalues of the infinitesimal generator of the right-hand side of (4) are in the left part of the complex plane, the stationary state **U **= 0 is asymptotically stable for equation (4). This result is difficult to prove because the spectrum (the main definitions for the spectrum of a linear operator are recalled in appendix A) of the infinitesimal generator neither reduces to the point spectrum (set of eigenvalues of finite multiplicity) nor is contained in a cone of the complex plane **C **(such an operator is said to be sectorial). The "principle of linear stability" is the fact that the linear stability of **U **is inherited by the state **V***^f ^*for the nonlinear equations (1) or (3). This result is stated in the corollaries 3.7 and 3.8.

Following [27-31], we note (**T**(*t*))_*t*≥0 _the strongly continuous semigroup of (4) on  (see definition A.3 in appendix A) and **A **its infinitesimal generator. By definition, if **U **is the solution of (4) we have **U***t *= **T**(*t*)*ϕ*. In order to prove the linear stability, we need to find a condition on the spectrum Σ(**A**) of **A **which ensures that **T**(*t*) → 0 as *t *→ ∞.

Such a "principle" of linear stability was derived in [29,30]. Their assumptions implied that Σ(**A**) was a pure point spectrum (it contained only eigenvalues) with the effect of simplifying the study of the linear stability because, in this case, one can link estimates of the semigroup **T **to the spectrum of **A**. This is not the case here (see proposition 3.4).

When the spectrum of the infinitesimal generator does not only contain eigenvalues, we can use the result in [[27], chapter 4, theorem 3.10 and corollary 3.12] for eventually norm continuous semigroups (see definition A.4 in appendix A) which links the growth bound of the semigroup to the spectrum of **A**:(5)

Thus, **U **is uniformly exponentially stable for (4) if and only if

We prove in lemma 3.6 (see below) that (**T**(*t*))_*t*≥0 _is eventually norm continuous. Let us start by computing the spectrum of **A**.

#### 3.1.1 Computation of the spectrum of A

In this section we use **L**_1 _for  for simplicity.

**Definition **3.1. *We define **for λ ∈ ***C ***by:*

*where ***J**(*λ*) *is the compact (it is a Hilbert-Schmidt operator, see [*[32]*, chapter X.2]) operator*

We now apply results from the theory of delay equations in Banach spaces (see [27,28,31]) which give the expression of the infinitesimal generator  as well as its domain of definition

The spectrum Σ(**A**) consists of those *λ *∈ **C **such that the operator Δ(*λ*) of  defined by

Δ(*λ*) = *λ*Id + **L**_0 _- **J**(*λ*) is non invertible. We use the following definition:

**Definition **3.2 (Characteristic values (CV)). *The characteristic values of ***A ***are the λs such that *Δ(*λ*) *has a kernel which is not reduced to 0, i.e., is not injective*.

It is easy to see that the CV are the eigenvalues of **A**.

There are various ways to compute the spectrum of an operator in infinite dimensions. They are related to how the spectrum is partitioned (for example continuous spectrum, point spectrum...). In the case of operators which are compact perturbations of the identity such as Fredholm operators, which is the case here, there is no continuous spectrum. Hence the most convenient way for us is to compute the point spectrum and the essential spectrum (see the appendix A). This is what we achieve next.

*Remark *1*. In finite dimension (i.e. ), the spectrum of ***A ***consists only of CV. We show that this is not the case here*.

Notice that most papers dealing with delayed neural field equations only compute the CV and numerically assess the linear stability (see [9,24,33]).

We now show that we can link the spectral properties of **A **to the spectral properties of **L***_λ_*. This is important since the latter operator is easier to handle because it acts on a Hilbert space. We start with the following lemma (see [34] for similar results in a different setting).

**Lemma 3.3**. *λ *∈ Σ*_ess_*(**A**) ⇔ *λ *∈ Σ*_ess _*(**L***_λ_*)

*Proof*. Let us define the following operator. If *λ *∈ **C**, we define  by , . From [[28], Lemma 34],  is surjective and it is easy to check that , see [[28], Lemma 35]. Moreover  is closed in  iif  is closed in , see [[28], Lemma 36].

Let us now prove the lemma. We already know that  is closed in  iff  is closed in . Also, we have , hence . It remains to check that .

Suppose that . There exist  such that . Consider . Because  is surjective, for all , there exists  satisfying . We write . Then  where *i.e*. .

Suppose that . There exist  such that . As  is surjective for all *i *= 1, ⋯, *N *there exists  such that . Now consider .  can be written  where . But  because . It follows that .

Lemma 3.3 is the key to obtain Σ(**A**). Note that it is true regardless of the form of **L **and could be applied to other types of delays in neural field equations. We now prove the important following proposition.

**Proposition 3.4. A ***satisfies the following properties:*

*1*. Σ*_ess_*(**A**) = Σ(-**L**_0_)

*2*. Σ(**A**) *is at most countable*.

*3*. Σ(**A**) = Σ(-**L**_0_) ∪ *CV*

*4. For λ *∈ Σ(**A**)\Σ(-**L**_0_), *the generalized eigenspace **is finite dimensional and *∃*k *∈ ℕ, 

*Proof*. 1. *λ *∈ Σ*_ess_*(**A**) ⇔ *λ *∈ Σ*_ess_*(**L***_λ_*) = Σ*_ess_*(-**L**_0 _+ **J**(*λ*)). We apply [[35], Theorem IV.5.26]. It shows that the essential spectrum does not change under compact perturbation. As  is compact, we find Σ*_ess_*(-**L**_0 _+ **J**(*λ*)) = Σ*_ess_*(-**L**_0_).

Let us show that Σ*_ess_*(-**L**_0_) = Σ(-**L**_0_). The assertion "⊂" is trivial. Now if *λ *∈ Σ(-**L**_0_), for example *λ *= -*l*_1_, then *λ*Id + **L**_0 _= *diag*(0, -*l*_1 _+ *l*_2_, ...).

Then  is closed but . Hence . Also , hence . Hence, according to definition A.7, .

2. We apply [[35], Theorem IV.5.33] stating (in its first part) that if Σ*_ess_*(**A**) is at most countable, so is Σ(**A**).

3. We apply again [[35], Theorem IV.5.33] stating that if Σ*_ess_*(**A**) is at most countable, any point in Σ(**A**)\Σ*_ess_*(**A**) is an isolated eigenvalue with finite multiplicity.

4. Because Σ*_ess_*(**A**) ⊂ Σ*_ess, Arino_*(**A**), we can apply [[28], Theorem 2] which precisely states this property.    □

**Figure 1:**
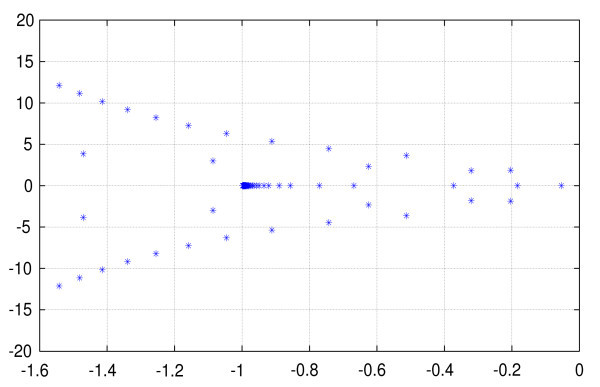
**Plot of the first 200 eigenvalues of A in the scalar case (*p *= 1, *d *= 1) and L_0 _= Id, *J*(*x*) = -1+1.5 cos(2*x*)**. The delay function *τ*(*x*) is the *π *periodic saw-like function shown in figure 2. Notice that the eigenvalues accumulate at *λ *= -1.

As an example, Figure 1 shows the first 200 eigenvalues computed for a very simple model one-dimensional model. We notice that they accumulate at *λ *= -1 which is the essential spectrum. These eigenvalues have been computed using TraceDDE, [36], a very efficient method for computing the CVs.

Last but not least, we can prove that the CVs are almost all, i.e. except for possibly a finite number of them, located on the left part of the complex plane. This indicates that the unstable manifold is always finite dimensional for the models we are considering here.

**Corollary 3.5**. *Card *Σ(**A**) ∩ {*λ *∈ ℂ, ℜ*λ >*-*l*} < ∞ *where l *= min*_i _l_i_*.

*Proof*. If *λ *= *ρ *+ *iω *∈ Σ(**A**) and *ρ >*-*l*, then *λ *is a CV *i.e. * stating that 1 ∈ Σ*_P_*((*λ*Id + **L**_0_)^-1^**J**(*λ*)) (Σ*_P _*denotes the point spectrum).

But  for *λ *big enough since  is bounded.

Hence, for *λ *large enough 1 ∉ Σ*_P_*((*λ*Id + **L**_0_)^-1^**J**(*λ*)), which holds by the spectral radius inequality. This relationship states that the CVs *λ *satisfying ℜ*λ >*-*l *are located in a bounded set of the right part of ℂ; given that the CV are isolated, there is a finite number of them.

#### 3.1.2 Stability results from the characteristic values

We start with a lemma stating regularity for (**T**(*t*))_*t *≥ 0_:

**Lemma 3.6**. *The semigroup *(**T**(*t*))_*t *≥ 0 _*of (4) is norm continuous on **for t > τ_m_*.

*Proof*. We first notice that -**L**_0 _generates a norm continuous semigroup (in fact a group)  on  and that  is continuous from  to . The lemma follows directly from [[27], Theorem VI.6.6].    □

Using the spectrum computed in proposition 3.4, the previous lemma and the formula (5), we can state the asymptotic stability of the linear equation (4). Notice that because of corollary 3.5, the supremum in (5) is in fact a max.

**Corollary 3.7 **(Linear stability). *Zero is asymptotically stable for (4) if and only if *maxℜΣ*_p_*(**A**) < 0.

We conclude by showing that the computation of the characteristic values of **A **is enough to state the stability of the stationary solution **V***^f^*.

**Corollary 3.8**. *If *maxℜΣ*_p_*(**A**) < 0, *then the persistent solution ***V***^f ^of (3) is asymptotically stable*.

*Proof*. Using **U **= **V **- **V***^f^*, we write (3) as . The function *G *is *C*^2 ^and satisfies *G*(0) = 0, *DG*(0) = 0 and . We next apply a variation of constant formula. In the case of delayed equations, this formula is difficult to handle because the semigroup **T **should act on non-continuous functions as shown by the formula , where *X*_0_(*θ*) = 0 if *θ <*0 and *X*_0_(0) = 1. Note that the function *θ *→ *X*_0_(*θ*)*G*(**U***_s_*) is not continuous at *θ *= 0.

It is however possible (note that a regularity condition has to be verified but this is done easily in our case) to extend (see [34]) the semigroup **T**(*t*) to the space . We note  this extension which has the same spectrum as **T**(*t*). Indeed, we can consider integral solutions of (4) with initial condition **U**_0 _in . However, as **L**_0_**U**_0_(0) has no meaning because *ϕ *→ *ϕ*(0) is not continuous in , the linear problem (4) is not well-posed in this space. This is why we have to extend the state space in order to make the linear operator in (4) continuous. Hence the correct state space is  and any function  is represented by the vector (*ϕ*(0), *ϕ*). The variation of constant formula becomes:

where *π*_2 _is the projector on the second component.

Now we choose *ω *= - maxℜΣ*_p_*(**A**)/2 > 0 and the spectral mapping theorem implies that there exists *M >*0 such that  and . It follows that  and from theorem 2.2, , which yields  and concludes the proof.    □

Finally, we can use the CVs to derive a sufficient stability result.

**Proposition 3.9**. *If **then ***V***^f ^is asymptotically stable for (3)*.

*Proof*. Suppose that a CV *λ *of positive real part exists, this gives a vector in the Kernel of Δ(*λ*). Using straightforward estimates, it implies that , a contradiction.    □

#### 3.1.3 Generalization of the model

In the description of our model, we have pointed out a possible generalization. It concerns the linear response of the chemical synapses, *i.e*., the lefthand side  of (1). It can be replaced by a polynomial in , namely , where the zeros of the polynomials *P_i _*have negative real parts. Indeed, in this case, when **J **is small, the network is stable. We obtain a diagonal matrix  such that **P**(0) = **L**_0 _and change the initial condition (as in the theory of ODEs) while the history space becomes  where *d_s _*+ 1 = max*_i _degP_i_*. Having all this in mind equation (1) writes(6)

Introducing the classical variable , we rewrite (6) as(7)

where  is the Vandermonde (we put a minus sign in order to have a formulation very close to 1) matrix associated to **P **and . It appears that equation (7) has the same structure as (1): , are bounded linear operators; we can conclude that there is a unique solution to (6). The linearized equation around a persistent states yields a strongly continuous semigroup  which is eventually continuous. Hence the stability is given by the sign of  where  is the infinitesimal generator of . It is then routine to show that

This indicates that the essential spectrum  of  is equal to ∪*_i_Root *(*P_i_*) which is located in the left side of the complex plane. Thus the point spectrum is enough to characterize the linear stability:

**Proposition 3.10**. *If  the persistent solution ***V***^f ^of (6) is asymptotically stable*.

Using the same proof as in [20], one can prove that  provided that .

**Proposition 3.11**. *If **then ***V***^f ^is asymptotically s*table.

### 3**.2 Principle of linear stability analysis via fixed point theory**

The idea behind this method (see [37]) is to write (4) as an integral equation. This integral equation is then interpreted as a fixed point problem. We already know that this problem has a unique solution in . However, by looking at the definition of the (Lyapunov) stability, we can express the stability as the existence of a solution of the fixed point problem in a smaller space . The existence of a solution in  gives the unique solution in . Hence, the method is to provide conditions for the fixed point problem to have a solution in ; in the two cases presented below, we use the Picard fixed point theorem to obtain these conditions. Usually this method gives conditions on the averaged quantities arising in (4) whereas a Lyapunov method would give conditions on the sign of the same quantities. There is no method to be preferred, rather both of them should be applied to obtain the best bounds.

In order to be able to derive our bounds we make the further assumption that there exists a *β >*0 such that:

Note that the notation ***τ ***^-*β *^represents the matrix of elements .

*Remark *2*. For example, in the 2D one-population case for , we have *0 ≤ *β <*1.

We rewrite (4) in two different integral forms to which we apply the fixed point method. The first integral form is obtained by a straightforward use the variation-of-parameters formula. It reads(8)

The second integral form is less obvious. Let us define

Note the slight abuse of notation, namely .

Lemma B.3 in appendix B.2 yields the upperbound . This shows that ∀*t, *.

Hence we propose the second integral form:(9)

We have the following lemma.

**Lemma 3.12**. *The formulation *(9) *is equivalent to (4)*.

*Proof*. The idea is to write the linearized equation as:(10)

By the variation-of-parameters formula we have:

We then use an integration by parts:

which allows us to conclude.    □

Using the two integral formulations of (4) we obtain sufficient conditions of stability, as stated in the following proposition:

**Proposition 3.13**. *If one of the following two conditions is satisfied:*

*1.  and there exist α *< 1, *β *> 0 *such that*

*where  represents the matrix of elements * .

*2. *.

*then ***V***^f ^is asymptotically stable for (3)*.

*Proof*. We start with the first condition.

The problem (4) is equivalent to solving the fixed point equation **U **= **P**_2_**U **for an initial condition . Let us define  with the supremum norm written , as well as

We define **P**_2 _on .

For all  we have  and (**P**_2_*ψ*)(0) = *ϕ*(0). We want to show that . We prove two properties.

**1. P**_2_*ψ ***tends to zero at infinity**.

Choose .

Using corollary.B.3, we have **Z**(*t*) → 0 as *t *→ ∞.

Let 0 <*T *<*t*, we also have

For the first term we write:

Similarly, for the second term we write

Now for a given *ε *> 0 we choose *T *large enough so that . For such a *T *we choose t* large enough so that  for *t *>t*. Putting all this together, for all *t *>t*:

From (9), it follows that **P**_2_*ψ *→ 0 when *t *→ ∞.

Since **P**_2_*ψ *is continuous and has a limit when *t *→ ∞ it is bounded and therefore .

**2. P**_2 _**is contracting on **

Using (9) for all *ψ*_1_ ,  we have

We conclude from Picard theorem that the operator **P**_2 _has a unique fixed point in .

There remains to link this fixed point to the definition of stability and first show that

where **U**(*t, ϕ*) is the solution of (4).

Let us choose *ε *> 0 and *M *≥ 1 such that . *M *exists because, by hypothesis, . We then choose *δ *satisfying(11)

and  such that . Next define

We already know that **P**_2 _is a contraction on  (which is a complete space). The last thing to check is , that is . Using lemma B.3 in appendix B.2:

Thus **P**_2 _has a unique fixed point **U***^ϕ,ε ^*in  which is the solution of the linear delayed differential equation *i.e*.

As **U***^ϕ,ε ^*(*t*) → 0 in  implies  in , we have proved the asymptotic stability for the linearized equation.

The proof of the second property is straightforward. If 0 is asymptotically stable for (4) all the CV are negative and corollary 3.8 indicates that **V***^f ^*is asymptotically stable for (3).

The second condition says that  is a contraction because .

The asymptotic stability follows using the same arguments as in the case of **P**_2_.

We next simplify the first condition of the previous proposition to make it more amenable to numerics.

**Corollary 3.14**. *Suppose that *∀*t *≥ 0, *with ε *> 0.

*If there exist α *< 1, *β *> 0 *such that *, *then ***V***^f ^is asymptotically stable*.

*Proof*. This corollary follows immediately from the following upperbound of the integral . Then if there exists *α *< 1, *β *> 0 such that , it implies that the condition 1. in proposition 3.13 is satisfied, from which the asymptotic stability of **V***^f ^*follows.    □

Notice that *ε *> 0 is equivalent to . The previous corollary is useful in at least the following cases:

• If  is diagonalizable, with associated eigenvalues/eigenvectors: , then  and .

• If **L**_0 _= *l*_0_I*d *and the range of  is finite dimensional:  where (*e_k_*)_*k*∈ℕ _is an orthonormal basis of , then  and . Let us write *J *= (*J_kl_*)_*k,l *= 1 ⋯ *N *_the matrix associated to  (see above). Then  is also a compact operator with finite range and . Finally, it gives .

• If  is self-adjoint, then it is diagonalizable and we can chose , *M_ε _*= 1

*Remark *3*. If we suppose that we have higher order time derivatives as in section 3.1.3, we can write the linearized equation as*(12)

*Suppose that **is diagonalizable then **where **and *. *Also notice that *. *Then using the same functionals as in the proof of proposition 3.13, we can find two bounds for the stability of a stationary state V^f^:*

• *Suppose that i.e. **V**^f ^is stable for the non-delayed equation where . If there exist α *< 1, *β *> 0 *such that *.

• .

To conclude, we have found an easy-to-compute formula for the stability of the persistent state **V***^f^*. It can indeed be cumbersome to compute the CVs of neural field equations for different parameters in order to find the region of stability whereas the evaluation of the conditions in Corollary 3.14 is very easy numerically.

The conditions in proposition 3.13 and corollary 3.14 define a set of parameters for which **V***^f ^*is stable. Notice that these conditions are only sufficient conditions: if they are violated, **V***^f ^*may still remain stable. In order to find out whether the persistent state is destabilized we have to look at the characteristic values. Condition 1 in proposition 3.13 indicates that if **V***^f ^*is a stable point for the non-delayed equation (see [18]) it is also stable for the delayed-equation. Thus, according to this condition, it is not possible to destabilize a stable persistent state by the introduction of small delays, which is indeed meaningful from the biological viewpoint. Moreover this condition gives an indication of the amount of delay one can introduce without changing the stability.

Condition 2 is not very useful as it is independent of the delays: no matter what they are, the stable point **V***^f ^*will remain stable. Also, if this condition is satisfied there is a unique stationary solution (see [18]) and the dynamics is trivial, i.e. converging to the unique stationary point.

### 3.3 Summary of the different bounds and conclusion

The next proposition summarizes the results we have obtained in proposition 3.13 and corollary 3.14 for the stability of a stationary solution.

**Proposition 3.15**. *If one of the following conditions is satisfied:*

*1. There exist ε >*0 *such that **and *α < 1, *β *> 0 *such that *,

*2. *

*then ***V***^f ^is asymptotically stable for (3)*.

The only general results known so far for the stability of the stationary solutions are those of Atay and Hutt (see for example [20]): they found a bound similar to condition 2 in proposition 3.15 by using the CVs, but no proof of stability was given. Their condition involves the L^1^-norm of the connectivity function **J **and it was derived using the CVs in the same way as we did in the previous section. Thus our contribution with respect to condition 2 is that, once it is satisfied, the stationary solution is asymptotically stable: up until now this was numerically inferred on the basis of the CVs. We have *proved *it in two ways, first by using the CVs, and second by using the fixed point method which has the advantage of making the proof essentially trivial.

Condition 1 is of interest, because it allows one to find the minimal propagation delay that does not destabilize. Notice that this bound, though very easy to compute, overestimates the minimal speed. As mentioned above, the bounds in condition 1 are sufficient conditions for the stability of the stationary state **V***^f^*. In order to evaluate the conservativeness of these bounds, we need to compare them to the stability predicted by the CVs. This is done in the next section.

## 4 Numerical application: Neural fields on a ring

In order to evaluate the conservativeness of the bounds derived above we compute the CVs in a numerical example. This can be done in two ways:

• Solve numerically the nonlinear equation satisfied by the CVs. This is possible when one has an explicit expression for the eigenvectors and periodic boundary conditions. It is the method used in [9].

• Discretize the history space  in order to obtain a matrix version **A***_N _*of the linear operator **A**: the CVs are approximated by the eigenvalues of **A***_N _*. Following the scheme of [36], it can be shown that the convergence of the eigenvalues of **A***_N _*to the CVs is in  for a suitable discretization of . One drawback of this method is the size of **A***_N _*which can be very large in the case of several neuron populations in a two-dimensional cortex. A recent improvement (see [38]), based on a clever factorization of **A***_N _*, allows a much faster computation of the CVs: this is the scheme we have been using.

The *Matlab *program used to compute the righthand side of (1) uses a *Cpp *code that can be run on multiprocessors (with the *OpenMP *library) to speed up computations. It uses a trapezoidal rule to compute the integral. The time stepper *dde23 *of *Matlab *is also used.

In order to make the computation of the eigenvectors very straightforward, we study a network on a ring, but notice that all the tools (analytical/numerical) presented here also apply to a generic cortex. We reduce our study to scalar neural fields Ω ⊂ ℝ and one neuronal population, *p *= 1. With this in mind the connectivity is chosen to be *homogeneous J*(*x*, *y*) = *J*(*x *- *y*) with *J even*. To respect topology, we assume the same for the propagation delay function *τ*(*x*, *y*).

We therefore consider the scalar equation with axonal delays defined on  with periodic boundary conditions. Hence  and *J *is also *π*-periodic.(13)

where the sigmoid *S*_0 _satisfies *S*_0_(0) = 0.

Remember that (13) has a Lyapunov functional when *c *= 0 and that all trajectories are bounded. The trajectories of the non-delayed form of (13) are heteroclinic orbits and no non-constant periodic orbit is possible.

We are looking at the local dynamics near the trivial solution *V ^f ^*= 0. Thus we study how the CVs vary as functions of the nonlinear gain *σ *and the "maximum" delay *c*. From the periodicity assumption, the eigenvectors of Δ(λ) are the functions cos(*nx*), sin(*nx*) which leads to the characteristic equation for the eigenvalues λ:(14)

where  is the Fourier Transform of *J *and . This nonlinear scalar equation is solved with the Matlab Toolbox TraceDDE (see [36]). Recall that the eigenvectors of **A **are given by the functions *θ *→ *e^λθ ^*cos(*nx*), *θ *→ *e^λθ ^*sin(*nx*) ∈  where λ is a solution of (14). A bifurcation point is a pair (*c*, *σ*) for which equations (14) have a solution with zero real part. Bifurcations are important because they signal a change in stability, a set of parameters ensuring stability is enclosed (if bounded) by bifurcation curves. Notice that if *σ*_0 _is a bifurcation point in the case *c *= 0, it remains a bifurcation point for the delayed system ∀*c*, hence ∀*c*, *σ *= *σ*_0_, 0 ∈ Σ(**A**). This is why there is a bifurcation line *σ *= *σ*_0 _in the bifurcation diagrams that are shown later.

**Figure 2:**
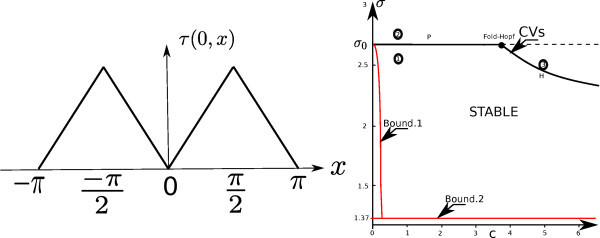
**Left: Example of a periodic delay function, the saw-function**. Right: plot of the CVs in the plane (*c*, *σ*), the line labelled *P *is the pitchfork line, the line labelled *H *is the Hopf curve. The two bounds of proposition 3.15 are also shown. Parameters are: *L*_0 _= Id, . The labels 1, 2, 3, indicate approximate positions in the parameter space (*c*, *σ*) at which the trajectories shown in Figure 3 are computed.

The bifurcation diagram depends on the choice of the delay function *τ*. As explained in section 2.1, we use *τ*(*x*, *y*) = *|x - y|_π_*, where the lower index *π *indicates that it is a *π*-periodic function. The bifurcation diagram with respect to the parameters (*c*, *σ*) is shown in the righthand part of Figure 2 in the case when the connectivity *J *is equal to . The two bounds derived in section 3.3 are also shown. Note that the delay-dependent bound is computed using the fact that  is self-adjoint. They are clearly very conservative. The lefthand part of the same figure shows the delay function *τ*.

The first bound gives the minimal velocity 1*/c *below which the stationary state might be unstable, in this case, even for smaller speed, the state is stable as one can see from the CV boundary. Notice that in the parameter domain defined by the 2 conditions bound.1. and bound.2., the dynamic is very simple: it is characterized by a unique and asymptotically stable stationary state, *V ^f ^*= 0.

**Figure 3:**
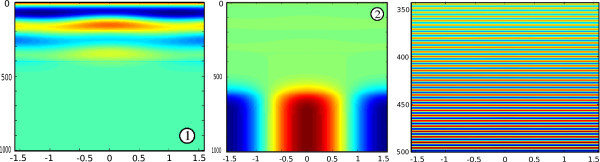
**Plot of the solution of (13) for different parameters corresponding to the points shown as 1, 2 and 3 in the righthand part of figure 2 for a random (in space) and constant (in time) initial condition, see text**. The horizontal axis corresponds to space, the range is . The vertical axis represents time.

In Figure 2 we show the dynamics for different parameters corresponding to the points labelled 1, 2 and 3 in the right-hand part of Figure 2 for a random (in space) and constant (in time) initial condition *ϕ *(see (1)). When the parameter values are below the bound computed with the CV, the dynamics converge to the stable stationary state *V ^f ^*= 0. Along the Pitchfork line labelled (P) in the right-hand part of Figure 2, there is a static bifurcation leading to the birth of new stable stationary states, this is shown in the middle part of Figure 3. The Hopf curve labelled (H) in the righthand part of Figure 2 indicates the transition to oscillatory behaviors as one can see in the righthand part of Figure 3. Note that we have not proved that the Hopf curve was indeed a Hopf bifurcation curve, we have just inferred numerically from the CVs that the eigenvalues satisfy the usual conditions for the Hopf bifurcation.

Notice that the graph of the CVs shown in the righthand part of Figure 2 features some interesting points, for example the Fold-Hopf point at the intersection of the Pitchfork line and the Hopf curve. It is also possible that the multiplicity of the 0 eigenvalue could change on the Pitchfork line (P) to yield a Bogdanov-Takens point.

These numerical simulations reveal that the Lyapunov function derived in [39] is likely to be incorrect. Indeed, if such a function existed, as its value decreases along trajectories, it must be constant on any periodic orbit which is not possible. However the third plot in Figure 3 strongly suggests that we have found an oscillatory trajectory produced by a Hopf bifurcation (which we did not prove mathematically): this oscillatory trajectory converges to a periodic orbit which contradicts the existence of a Lyapunov functional such as the one proposed in [39].

Let us comment on the tightness of the delay-dependent bound: as shown in proposition 3.13, this bound involves the maximum delay value *τ_m _*and the norm , hence the specific shape of the delay function, *i.e*. , is not completely taken into account in the bound. We can imagine many different delay functions with the same values for *τ_m _*and  that will cause possibly large changes to the dynamical portrait. For example in the previous numerical application the singularity *σ *= *σ*_0_, corresponding to the fact that 0 ∈ Σ*_p_*(**A**), is independent of the details of the shape of the delay function: however for specific delay functions, the multiplicity of this 0-eigenvalue could change as in the Bogdanov-Takens bifurcation which involves changes in the dynamical portrait compared to the pitchfork bifurcation. Similarly, an additional purely imaginary eigenvalue could emerge (as for *c *≈ 3.7 in the numerical example) leading to a Fold-Hopf bifurcation. These instabilities depend on the expression of the delay function (and the connectivity function as well). These reasons explain why the bound in proposition 3.13 is not very tight.

This suggests another way to attack the problem of the stability of fixed points: one could look for connectivity functions  which have the following property: for all delay function ***τ***, the linearized equation (4) does not possess 'unstable solutions', *i.e*. for all delay function ***τ***, ℜΣ*_p_*(**A**) < 0. In the literature (see [40,41]), this is termed as the *all-delay *stability or the *delay-independent *stability. These remain questions for future work.

## 5 Conclusion

We have developed a theoretical framework for the study of neural field equations with propagation delays. This has allowed us to prove the existence, uniqueness, and the boundedness of the solutions to these equations under fairly general hypotheses.

We have then studied the stability of the stationary solutions of these equations. We have proved that the CVs are sufficient to characterize the linear stability of the stationary states. This was done using the semigroups theory (see [27]).

By formulating the stability of the stationary solutions as a fixed point problem we have found delay-dependent sufficient conditions. These conditions involve all the parameters in the delayed neural field equations, the connectivity function, the nonlinear gain and the delay function. Albeit seemingly very conservative they are useful in order to avoid the numerically intensive computation of the CV.

From the numerical viewpoint we have used two algorithms [36,38] to compute the eigenvalues of the linearized problem in order to evaluate the conservativeness of our conditions. A potential application is the study of the bifurcations of the delayed neural field equations.

By providing easy-to-compute sufficient conditions to quantify the impact of the delays on neural field equations we hope that our work will improve the study of models of cortical areas in which the propagation delays have so far been somewhat been neglected due to a partial lack of theory.

### A Operators and their spectra

We recall and gather in this appendix a number of definitions, results and hypotheses that are used in the body of the article to make it more self-sufficient.

**Definition **A.1. *An operator T *∈ ⋯(*E*, *F*), *E*, *F being Banach spaces, is closed if its graph is closed in the direct sum E *⊕ *F*.

**Definition **A.2. *We note **the operator norm of a bounded operator **, i.e*.

*It is known, see e.g. *[35]*, that*

**Definition **A.3. *A semigroup *(**T**(*t*))_*t*≥0 _*on a Banach space E is strongly continuous if *∀*x *∈ *E, t *→ *T *(*t*)*x is continuous from *ℝ_+ _*to E*.

**Definition **A.4. *A semigroup *(**T**(*t*))_*t*≥0 _*on a Banach space E is norm continuous if t *→ *T *(*t*) *is continuous from *ℝ_+ _*to L*(*E*)*. It is said eventually norm continuous if it is norm continuous for t > t_0 _≥ 0*.

**Definition **A.5. *A closed operator **of a Banach space E is Fredholm if **and **are finite and **is closed in E*.

**Definition **A.6. *A closed operator **of a Banach space E is semi-Fredholm if * or *is finite and **is closed in E*.

**Definition **A.7. *If **is a closed operator of a Banach space E the essential spectrum *Σ*_ess_*(*T *) *is the set of λs in *ℂ *such that λ*Id - *T is not semi-Fredholm i.e. either **is not closed or **is closed but *.

*Remark 4. *[28]*uses the definition: λ *∈ Σ*_ess,Arino_*(*T*) *if at least one of the following holds: **is not closed or **is infinite dimensional or λ is a limit point of *Σ(*T*). *Then *

### B The Cauchy problem

#### B.1 Boundedness of solutions

We prove lemma B.2 which is used in the proof of the boundedness of the solutions to the delayed neural field equations (1) or (3).

**Lemma B.1**. *We have **and *.

*Proof*. • We first check that **L**_1 _is well defined: if  then *ψ *is measurable (it is Ω-measurable by definition and [0, *τ*]-measurable by continuity) on Ω × [-*τ_m_*, 0] so that the integral in the definition of **L**_1 _is meaningful. As *τ *is continuous, it follows that is measurable on Ω^2^. Furthermore (*ψ^d^*)^2 ^∈ **L**^1^(Ω^2^,ℝ^*p *× *p*^).

• We now show that . We have for , .

With Cauchy-Schwartz:(15)

Nothing that

 we obtain

and **L**_1 _is continuous.    □

**Lemma B.2**. *We have *.

*Proof*. By the Cauchy-Schwarz inequality and lemma B.1:

 because *S *is bounded by 1.    □

### B.2 Stability

In this section we prove lemma B.3 which is central in establishing the first sufficient condition in proposition 3.13.

**Lemma B.3**. *Let β >*0 *be such that **τ **^-β ^*∈**L**^2^(Ω^2 ^, ℝ^*p *× *p*^)). *Then we have the following bound*.

*Proof*. We have:(16)

and if we set , we have:

 and from the Cauchy-Schwartz inequality:

Again, from the Cauchy-Schwartz inequality applied to :(17)

Then, from the discrete Cauchy-Schwartz inequality:(18)

which gives as stated:

      □

and allows us to conclude.

## Competing interests

The authors declare that they have no competing interests.
